# In utero, lactational, or peripartal fluoxetine administration has differential implications on the murine maternal skeleton

**DOI:** 10.14814/phy2.15837

**Published:** 2023-10-09

**Authors:** Hannah P. Fricke, Chandler J. Krajco, Molly J. Perry, Maggie A. Reisner, Lauren J. Brettingen, Lella A. Wake, Julia F. Charles, Laura L. Hernandez

**Affiliations:** ^1^ Endocrinology and Reproductive Physiology Program University of Wisconsin‐Madison Madison Wisconsin USA; ^2^ Department of Dairy Science University of Wisconsin‐Madison Madison Wisconsin USA; ^3^ Departments of Orthopedics and Medicine Brigham and Women's Hospital Boston Massachusetts USA

**Keywords:** bone, lactation, serotonin, SSRI

## Abstract

The peripartal period is marked by alterations in calcium metabolism to accommodate for embryonic skeletal mineralization and support bone development of offspring in early life, and serotonin plays a critical role in modulating peripartal bone remodeling. Selective serotonin reuptake inhibitors (SSRIs) are commonly used as first‐line treatment for psychiatric illness during pregnancy and the postpartum period and considered safe for maternal use during this time frame. In order to evaluate the effect of peripartal alterations of the serotonergic system on maternal skeletal physiology, we treated dams with the SSRI fluoxetine during gestation only, lactation only, or during the entire peripartal period. Overall, we found a low dose of fluoxetine during gestation only had minimal impacts on maternal bone at weaning, but there were implications on maternal skeleton at weaning when dams were exposed during lactation only or during the entire peripartal period. We found that these effects were differential between female mice dosed lactationally or peripartally, and there were also impacts on maternal mammary gland at weaning in both of these groups. Though SSRIs are largely considered safe maternally during the peripartal period, this study raises the question whether safety of SSRIs, specifically fluoxetine, during the peripartal period should be reevaluated.

## 1 INTRODUCTION

The World Health Organization recommends exclusive breastfeeding during the first 6 months of life, emphasizing the importance of breastfeeding to both parent and offspring (*Infant and young child feeding*, 2021). During the postpartum period, an estimated 10%–15% of the population experiences postpartum depression, and selective serotonin reuptake inhibitors (SSRIs) are often used as the preferred treatment for pregnant and lactating individuals and the general population (Burt & Stein, 2002; Cooper et al., 2007; Kroska & Stowe, 2020). There is evidence from both human and animal studies that SSRIs are associated with decreased bone mass, which may be attributed to the role of serotonin in bone remodeling (Bonnet et al., 2007; Ortuño et al., 2016; Rabenda et al., 2013; Tsapakis et al., 2012; Warden et al., 2005). Further, in humans, 6 months of exclusive breastfeeding has been correlated with a 6%–10% decrease in maternal bone mineral density (BMD) (Kovacs, 2016). It was previously thought that maternal BMD was restored 12 months after weaning in a normal physiological state, but the validity of this belief has recently been challenged (Bjørnerem et al., 2017; Hwang et al., 2016; Kovacs, 2016; Wysolmerski, 2010). Lactation and SSRIs are independently associated with decreased bone mass, and the use of SSRIs during the peripartal period may result in sustained maternal bone loss (Weaver et al., 2018). Previously, we have demonstrated that administration of the SSRI fluoxetine (Prozac™) during the peripartal period resulted in a negative impact on maternal bone mass postweaning in mice (Weaver et al., 2018).

Classically considered antidepressants, SSRIs are also used to treat many serotonin‐related disorders including anxiety disorders, obsessive‐compulsive disorder, and post‐traumatic stress disorder (Nutt et al., 1999). Fluoxetine was the first SSRI introduced in the United States in 1987 and is the second most commonly prescribed antidepressant, surpassed only by the SSRI sertraline (Zoloft™) (Fuentes et al., 2018; Wong et al., 2005). The SSRI class of antidepressants exert their action by blocking the serotonin receptor, SERT, and impeding the reuptake of serotonin into the presynaptic neurons (Sohel et al., 2021). Though SSRIs are designed to target serotonin signaling in the brain, they also impact serotonin in the periphery, as SERT is genetically identical throughout the body. Despite the importance of central serotonin, the overwhelming majority of serotonin, approximately 95%, resides in the gut, primarily synthesized by the enterochromaffin cells, and circulating serotonin is taken up into platelets by SERT to be transported throughout the body (Gershon & Tack, 2007). By blocking peripheral SERT, SSRIs can upregulate serotonin signaling in the periphery, increase serotonin synthesis, and decrease serotonin degradation (Marshall et al., 2014).

In pregnancy, maternal calcium absorption increases, but this is not true during lactation, and so calcium is sourced from bone stores in order to provide sufficient calcium for the growing offspring (Kovacs, 2016; Shenolikar, 1970; Specker et al., 1994). In order to accommodate the calcium demands of the offspring, the lactating mammary gland becomes an important mediator of calcium metabolism and bone remodeling. During this time frame, the mammary gland signals to bone in a serotonin‐dependent endocrine fashion to regulate bone metabolism via the release of parathyroid hormone‐related protein (PTHrP) into circulation, which then acts on bone to increase osteoclast‐driven bone resorption and osteocytic osteolysis (Liu et al., 2012; Miyamoto et al., 2019; Qing et al., 2012; Sowers et al., 1996; VanHouten et al., 2003). This, in turn, liberates calcium into circulation to return to the mammary gland for milk production. Serotonin drives the release of PTHrP in the mammary gland by inducing the canonical hedgehog signaling pathway and subsequently altering the sonic hedgehog promoter methylation patterns, resulting in the activation of PTHrP signaling (Laporta et al., 2014).

The role of serotonin in bone remodeling was first established in 2001 when two separate studies found evidence of the presence of SERT and serotonin receptors in bone cells, but the complete role of serotonin in bone metabolism is not yet fully understood (Bliziotes et al., 2001; Ducy & Karsenty, 2010; Weissman et al., 2004). Modulation of peripheral serotonin signaling by SSRIs may explain the association between SSRIs and decreased bone mass. However, the effect on bone may differ depending on dosage, specific SSRIs, the age at which the SSRI is administered, and length of dosing. Fluoxetine, the first SSRI introduced in the United States, is the second most commonly prescribed antidepressant today in the general population and is commonly used among peripartal individuals (Fuentes et al., 2018; Wong et al., 2005). In rodents, fluoxetine has been shown to have a significant impact on bone, whereas sertraline and escitalopram had little to no impact (Kumar et al., 2018; Sheftel et al., 2022). Further, it has previously been reported that there are different bone phenotypes associated with the length of fluoxetine treatment in mice; treatment for 3 weeks resulted in an increased bone mass while treatment for 6 weeks resulted in bone mass loss (Ortuño et al., 2016). Previously, we have established that a high dose of fluoxetine during pregnancy and lactation had long‐term impacts on maternal bone, and that this primarily occurred through stimulation of PTHrP in the mammary gland (Weaver et al., 2018). The aim of this study was to expand upon our previous findings and examine the impact of a low dose of fluoxetine on maternal trabecular bone fraction (BV/TV) at weaning and 3 months postweaning when administered during gestation, lactation, or both gestation and lactation.

## 2 MATERIALS AND METHODS

### 2.1 Animals

All experiments were approved by the Research Animal Care and Use Committee at the University of Wisconsin–Madison and were performed under protocol number A005789‐R01‐A03. Female C57BL/6 mice were obtained from Jackson Laboratories at 5 weeks of age ± 3 days (stock #000664, Jackson Laboratories, Bar Harbor, ME). Until the first day of pregnancy, at which point they were housed separately, mice were housed in groups in an environmentally controlled facility for biological research in the Biochemistry department vivarium at the University of Wisconsin–Madison. Mice were maintained at a temperature of 25°C and a humidity of 50%–60% on a 12‐h light/dark cycle with food (Envigo‐Teklad #2018) and water access ad libitum. Eight to ten mice/group were enrolled per group for the assessment of the various outcomes measured. Two‐way ANOVA with a repeated measures design, and a Tukey's multiple comparison test were used to analyze serum calcium, serotonin, PTHLH, C‐terminal telopeptide fragments of type‐I collagen crosslinks, homocysteine, and milk yield to compare treatments over time. One‐way ANOVA with a Tukey's post hoc test was used to compare all measurements at weaning, and bone parameters at individual time points. With 10 animals per group, we have 80% power with an alpha of 0.05 to detect a significant difference of 3‐fold changes in gene expression/metabolite concentrations with a standard deviation of 1.2 between groups.

Beginning at 6 weeks of age, female mice were mated overnight with a male C57BL/6 mouse also obtained from Jackson Laboratories. Pregnancy was determined by the presence of a vaginal plug, at which point the female mice were housed individually. On the first day of pregnancy (E0), mice were randomly assigned to receive sterile saline or 2 mg/kg body weight of fluoxetine hydrochloride (catalog no. S6319; Sigma‐Aldrich, St. Louis, MO, USA) reconstituted in sterile saline daily. When accounting for physiological differences between rodents and humans, this dose approximates a 60 kg human taking 10 mg of fluoxetine per day (Research C for DE, 2018; Shin et al., 2010). Treatment was administered via intraperitoneal injection between 0800 and 0900 daily. The weight of the dams and the number and weight of the pups were recorded daily at the time of injection from E0 to the end of lactation (D21). The litters were not standardized due to the effect of fluoxetine on early pup mortality as reported previously (Domingues et al., 2022).

#### 2.1.1 Experiment 1 (Figure 1)

**FIGURE 1:**
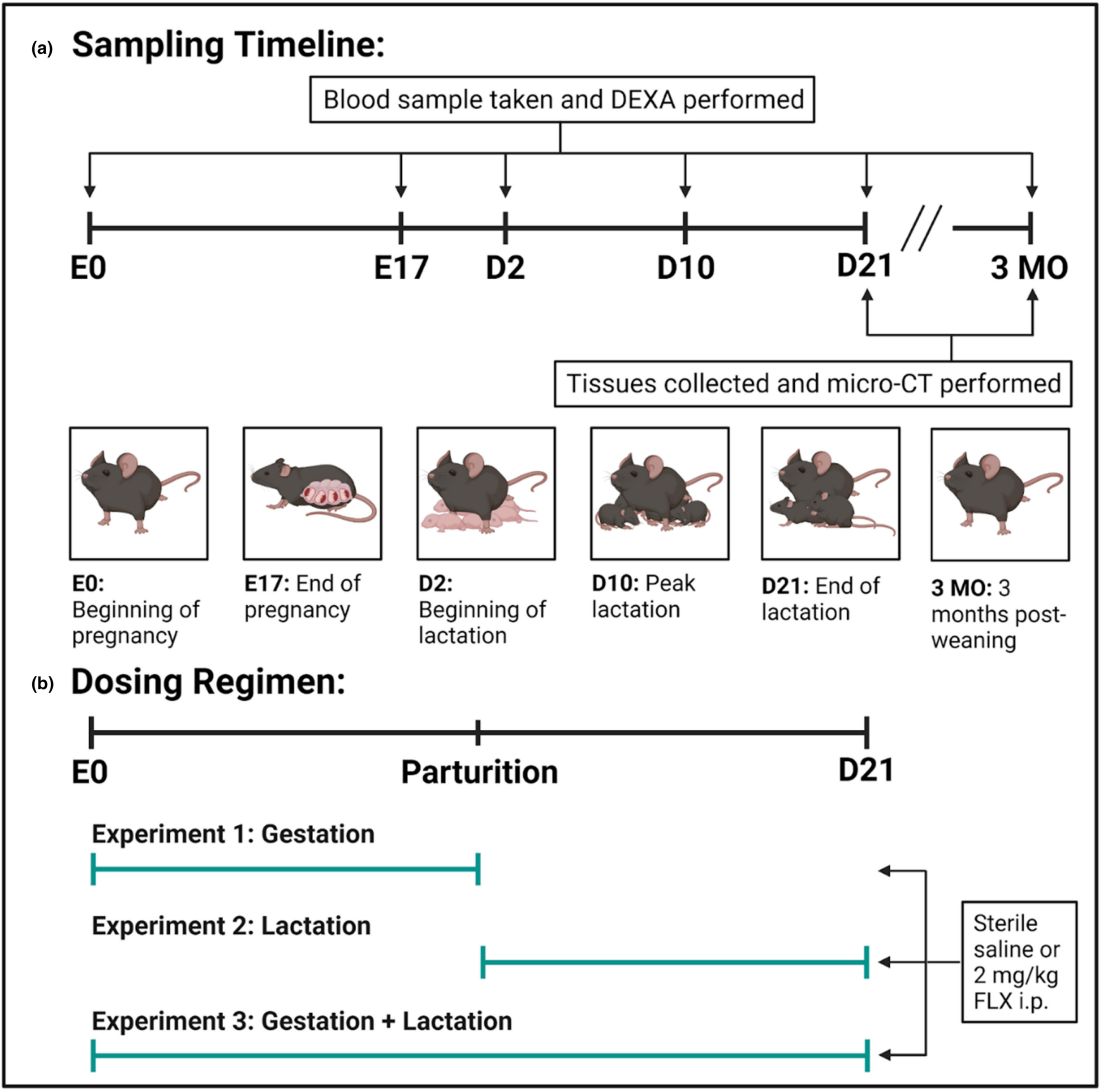
Sampling timeline and dosing regimen of the study. (a) Sampling timeline. Blood was collected on the date of conception (E0), the end of gestation (E17), the beginning of lactation (D2), peak lactation (D10), the end of lactation (D21), and 3 months postweaning (3 MO). Bone mineral density (BMD) was measured via dual‐energy X‐ray absorptiometry (DXA) at the same time points. At the terminal endpoints, D21 and 3 MO, the bone and mammary glands were harvested and micro‐computed tomography (micro‐CT) was performed. (b) Dosing regimen. C57BL/6J dams were administered sterile saline or 2 mg/kg fluoxetine during gestation only (date of conception through the end of pregnancy), lactation only (first day of lactation through the end of lactation), or throughout gestation and lactation (date of conception through the end of lactation).

The first experiment was designed to examine the effect of fluoxetine administration during gestation only, in order to determine the gestational effects of SSRI treatment. On the first day of pregnancy, the dams were randomly assigned to receive either sterile saline (*n* = 16) or 2 mg/kg fluoxetine (*n* = 17) daily from E0 through the end of pregnancy. The dams were then either collected at weaning (saline: *n* = 7; fluoxetine: *n* = 8) or were aged out to 3 months postweaning (saline: *n* = 9; fluoxetine: *n* = 9).

#### 2.1.2 Experiment 2 (Figure 1 )

The second experiment was designed to examine the effect of fluoxetine administration during lactation only, and determine the effects of SSRI during lactation only. On the first day of lactation, the dams were randomly assigned to receive either sterile saline (*n* = 17) or 2 mg/kg fluoxetine (*n* = 18) daily from the first day of lactation through D21. The dams were then either collected at weaning (saline: *n* = 8; fluoxetine: *n* = 10) or were aged out to 3 months postweaning (saline: *n* = 9; fluoxetine: *n* = 8).

#### 2.1.3 Experiment 3 (Figure 1)

The final experiment was designed to examine the effect of fluoxetine administration during the entire peripartal period, from the beginning of gestation through the end of lactation. On the first day of pregnancy, the dams were randomly assigned to receive either sterile saline (*n* = 16) or 2 mg/kg fluoxetine (*n* = 19) daily from E0 through D21. The dams were then either collected at weaning (saline: *n* = 8; fluoxetine: *n* = 10) or were aged out to 3 months postweaning (saline: *n* = 8; fluoxetine: *n* = 9).

### 2.2 Sample collection

Blood samples were taken between 1400 and 1500 after a 6–7 h fasting period. Blood was collected into Eppendorf tubes from the maxillary vein on E0, E17, D2, D10, D21, and 3 MO. The blood was centrifuged at 1500 *g* at 4°C for 20 min to isolate serum, and serum was stored at −80°C until the time of assay.

At D21 or 3 MO, the dams were euthanized via carbon dioxide inhalation, followed by cervical dislocation to ensure clinical death. The left femur was collected for micro‐CT and was fixed in 70% ethanol until micro‐CT analysis. The right femur and lower right mammary gland were harvested, snap‐frozen in liquid nitrogen, and stored at −80°C until the time of assay. The lower left mammary gland was fixed overnight in a histological cassette at 4°C in 4% paraformaldehyde and then placed in 70% ethanol until embedded in paraffin.

### 2.3 DXA analysis

Bone densitometry and body composition of the exposed dams were measured using dual‐energy X‐ray absorptiometry (DXA) via a PIXImus2 Mouse Densitometer (GE Medical Systems, Madison, WI) as previously described (Lee et al., 2014). Quality control measurements were performed with a phantom before each session, and the mice were anesthetized during each measurement via isoflurane inhalation. Measurements were taken at 6 weeks of age as a baseline measurement, then at E17, D2, D10, D21, and 3 MO. Analysis of the scans was performed with the Lunar Piximus Software using autothresholding, and bone mineral density (BMD) of the femur and total body was measured.

### 2.4 Micro‐CT analysis

The femurs of the exposed dams were analyzed by micro‐computed tomography (micro‐CT) using a Scanco Medical μCT 35 system and integrated Scanco reconstruction software with an isotropic voxel size of 7 μm. Scans were conducted in 70% ethanol using an X‐ray tube potential of 55kVp, an X‐ray intensity of 0.145 mA, and an integration time of 400 ms. Digital calipers were used to measure femoral length. Cancellous bone analysis was measured via a selected region beginning 0.14 mm proximal to the growth plate and extending 1.4 mm proximally. Cortical parameters were calculated via a selected region centered at the midpoint of the femur and 0.6 mm in length. Cortical and trabecular bone were distinguished via a semiautomated contouring approach. The region of interest was selected using a global threshold that set the bone/marrow cutoff at 512 mgHA/cm^3^ for trabecular bone and 871.8 mgHA/cm^3^ for cortical bone. The three‐dimensional microstructural properties of the bone, which include the bone volume fraction (BV/TV), trabecular thickness (Tb.Th), trabecular number (Tb.N.), trabecular separation (Tb.Sp.), midshaft bone volume fraction (M.BV/TV), and cortical thickness (C.Th) were calculated with software supplied by the manufacturer and were reported according to consensus guidelines on rodent micro‐CT (Bouxsein et al., 2010).

### 2.5 Assays

Serum serotonin concentrations were measured using the Beckman Coulter Enzyme Immunoassay Kit (catalog no. IM1749; Beckman Coulter, Vršovice, Czech Republic) per the manufacturer's instructions. Serum samples were diluted 1:200 to fit within the standard curve. Serum calcium concentrations were measured via Cayman Chemicals Calcium Assay Kit (catalog no. 701220; Cayman Chemicals, Ann Arbor, MI) per manufacturer's instructions. Samples were diluted 1:2 to fit within the standard curve. Serum procollagen I intact N‐terminal (P1NP) concentrations were measured via Immunodiagnostics Systems enzyme immunoassay (catalog no. AC‐33F1; Immunodiagnostics Systems, Tyne and Wear, United Kingdom) per manufacturer's instructions. Samples were diluted 1:10 to fit within the standard curve. Serum collagen type 1 cross‐linked C‐telopeptide (CTX) concentrations were measured via RatLaps™ (CTX‐I) Immunodiagnostics Systems enzyme immunoassay (catalog no. AC‐06F1; Immunodiagnostics Systems, Tyne and Wear, United Kingdom) per manufacturer's instructions. All assays had an intra‐assay CV of <15% and interassay CV of <15%.

### 2.6 Mammary gland and femur RNA and RT‐qPCR

Total RNA was extracted from the mammary gland and femur using TRI‐Reagent (catalog no. NC9330796; Molecular Research, Cincinnati, OH) and RNA was reverse transcribed (1 μg) to cDNA via the Applied Biosystems High Capacity cDNA Reverse Transcription Kit (catalog no. 4368814; Applied Biosystems, Foster City, CA). Quantitative RT‐PCR was performed using the CFX96 Touch Real‐Time PCR Detection System (Bio‐Rad Laboratories, Rodeo, CA), and reaction mixtures and cycling conditions were performed as previously described (Laporta et al., 2013). Primers were designed to span exon–exon junctions with an optimal annealing temperature of 60°C, and amplification efficiencies were accepted within 95%–105%. The presence of a single temperature dissociation peak determined primer specificity. The primer sequences are listed in Table 1. In the mammary gland, the housekeeping parameter was the geometric mean of *Rps15*, *Rps9*, *K8*, and *K14*. In the femur, the housekeeping parameter was the geometric mean of *Rps15* and *Hprt1*. Analysis was conducted using the 2^−ΔΔCT^ method (Livak & Schmittgen, 2001).

**TABLE 1:** Primer sequences used for RT‐qPCR.

Gene	Forward primer 5′ → 3′	Reverse primer 3′ → 5′
*Casp3*	CCAAATGAGAAAGCTGTCAGG	TTGAGGTAGCTGCACTGTGG
*Ccnd1*	TGATTCTGGCACATTCTTGC	TCACCTCTTCCCTCACATCC
*Gli1*	GGCAGGGAAGAGAGCAGACT	ACTGCCTGCTGGGGAGTG
*Hprt1*	CTGGTGAAAAGGACCTCTCG	AACTTGCGCTCATCTTAGGC
*Krt8*	ATCGAGATCACCACCTACCG	AAGCCAGGGCTAGTGAGTCC
*Krt14*	TCTTGGCGGTGGTATTGGTGAT	CAGGCTCTGCTCCGTCTCAAACT
*M‐csf*	CGAATGTTCTCCCACTTCCT	TGGACAATCAAAGGCTGAGG
*Mcp1*	CCAAAGAAGCTGTAGTTTTTG	GGTTCCGATCCAGGTTTTTA
*Mmp13*	CCGAACTTAACTTACAGGATTG	GGTGTCACTCAGACCAGACC
*Opg*	AAGCTGGAACCCCAGAGC	GTGCTGCACTTCGTGTGTTT
*Orai1*	ACCCCACGAGCGCATGCATC	GCTTGGTGGGGCTTGGCTGT
*Pmca2*	ACGTATGGGGACACTGAAGC	TTGCCCAAAAATCTGTTTCC
*Pthlh*	TTCCTGCTCAGCTACTCCGT	GATGGACTTGCCCTTGTCAT
*Rank*	CAGGACAGGGCTGATGAGAG	CCGCTAGAGATGAACGTGGA
*Rankl*	GGAGGATGAAACAAGCCTTTG	ACATCCAACCATGAGCCTTC
*Rsp9*	GGAGACCCTTCGAGAAGTCG	GGGGATCCTTCTCGTCTAGC
*Rsp15*	TTGAGAAAGGCCAAAAAGGA	GTTGAAGGTCTTGCCGTTGT
*Shh*	CTCCGATGTGTTCCGTTACC	GCCTGGCTCTTTCTCTTCCT
*Tnfα*	AGACCCTCACACTCAGATCAT	TCAGCCACTCCAGCTGCT
*Tph1*	TTCACCATGATTGAAGACAAC	TCCGACTTCATTCTCCAAGG
*Trap*	CGACAAGAGGTTCCAGGAGA	TGCCAAGGTGATCATGGTTT

Abbreviations: *Casp3*, caspase‐3; *Ccnd1*, cyclin D1; *Gli1*, GLI family zinc finger 1; *Hprt1*, hypoxanthine phosphoribosyltransferase 1; *Krt8*, keratin 8; *Krt14*, keratin 14; *M‐csf*, colony stimulating factor 1; *Mcp1*, monocyte chemoattractant protein‐1; *Mmp13*, matrix metallopeptidase 13; *Opg*, osteoprotegrin; *Orai1*, calcium release‐activated calcium modulator 1; *Pmca2*, plasma membrane Ca^2+^ ATPase 1; *Pthlh*, parathyroid hormone like hormone; *Rank*, receptor activator of nuclear factor κΒ; *Rankl*, receptor activator of nuclear factor κΒ ligand; *Rsp9*, 40S ribosomal protein S9; *Rsp15*, 40S ribosomal protein S15; *Shh*, sonic hedgehog; *Tnfα*, tumor necrosis factor alpha; *Tph1*, tryptophan hydroxylase 1; *Trap*, tartrate‐resistant acid phosphatase.

### 2.7 Mammary gland histology and immunofluorescence

Mammary glands were sectioned and stained with hematoxylin and eosin (H&E). Sections were also deparaffinized and processed for immunofluorescence with the following antibodies: TPH1 (Abcam, #ab228588; 1:100) and PCNA (Santa Cruz Biotechnology #sc‐56; 1:100). The secondary antibodies used were Alexa Fluor 594 Goat Anti‐Rabbit IgG (Life Technologies, #A11037; 1:250) and Alexa Fluor 488 Goat Anti‐Mouse IgG (Life Technologies, #A11001; 1:250) and were incubated for 1 h at room temperature. DAPI, dilactate (4′, 6‐diamidino‐2‐phenylindole, dilactate) (Invitrogen, #D3571; 300 nM final concentration) was used to visualize nuclei. All images were captured via QuPath‐0.3.2 software on a Zeiss Axio Vert. One microscope at 20x.

### 2.8 Statistics

All statistical analyses were conducted using GraphPad Prism 9 (Version 9.5.1). Analyses between the treatment groups without the effect of time were performed using a Student's unpaired two‐sided *t‐*test. When data were not normally distributed, a Kruskal–Wallis test was performed for nonparametric data. Analyses with multiple time points were conducted using a two‐way ANOVA with Tukey's multiple comparisons test to detect differences between treatment groups. Outliers were determined using the Prism ROUT method analysis. For analyses, differences among means were considered significant if *p* < 0.05 or a tendency if 0.05 < *p* < 0.1. All values are reported as mean ± SD.

## 3 RESULTS

### 3.1 During gestation only, fluoxetine administration does not have long‐term or short‐term impact on bone

**FIGURE 2:**
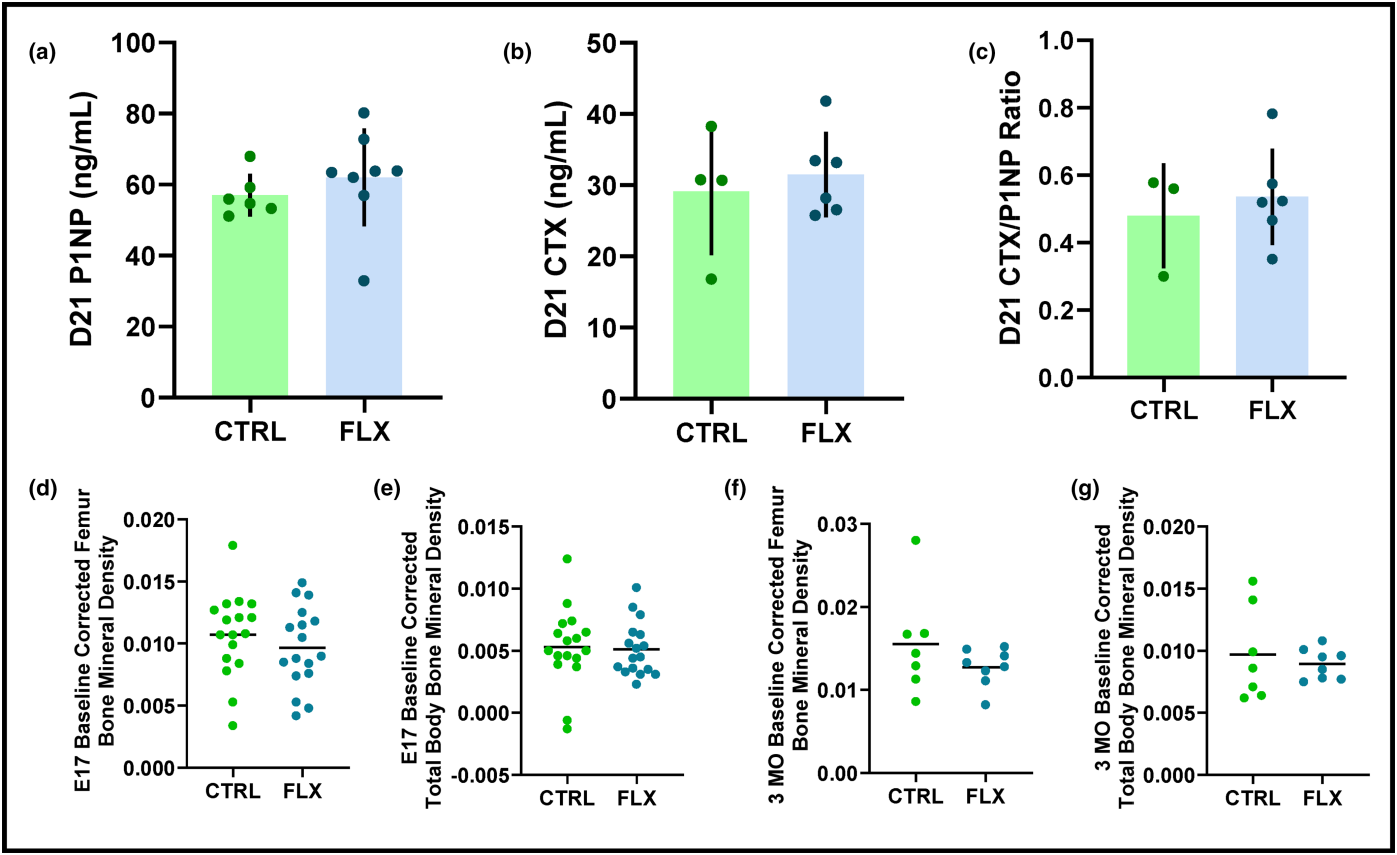
Fluoxetine during gestation only did not have significant impacts on bone remodeling. C57Bl/6J dams were administered sterile saline (*n* = 16) or 2 mg/kg fluoxetine (*n* = 17) daily from the beginning of gestation (E0) through the end of pregnancy. The dams were either harvested at weaning (D21) (*n* = 7 CTRL; *n* = 9 FLX) or were aged out to 3 months postweaning (3 MO) (*n* = 8 CTRL; *n* = 9 FLX). Blood was collected and serum was isolated from the dams 6 h after treatment on E0, E17, and D21. Serum (a) P1NP, (b) CTX, and (c) the CTX/P1NP ratio at D21 are shown. (d–g). Dual‐energy X‐ray absorptiometry (DXA) was used to measure bone mineral density (BMD) of the femur and the total body at the end of gestation and 3 months postweaning. (d) The BMD of the femur at the end of gestation relative to E0. (e) The BMD of the total body at the end of gestation relative to E0. (f) The BMD of the femur 3 months postweaning relative to D21. (g) The BMD of the total body 3 months postweaning relative to D21. *p* < 0.05 is considered significant and 0.1 < *p* < 0.05 is considered a tendency.

In order to determine the impact fluoxetine administration during gestation had on maternal bone, circulating markers of bone remodeling were measured in the dam serum at the end of lactation. There were no significant differences in circulating P1NP, CTX, or the P1NP/CTX ratio (Figure 2; Figure 2a–c). At the end of gestation, DXA analysis was used to measure the change in BMD of both the femur and the total body compared to the beginning of gestation (Figure 2; Figure 2d,e). There were no significant differences between either group. At 3 months postweaning (3 MO), DXA was used again to measure the change in BMD of the femur and total body compared to the end of lactation (Figure 2f,g). Similarly, there were no significant differences at either site.

Micro‐CT analysis was used to investigate the characteristics of the cortical and trabecular bone (Table 2). In the cortical bone, there was a significant increase in the cortical thickness at 3 months postweaning compared to D21 (*p* < 0.0001) and a tendency for there to be an increase in the FLX animals compared to the controls (*p* < 0.1). The periosteal perimeter was decreased at 3 MO compared to D21 (*p* < 0.0001), and there was a significant interaction between treatment and time (*p* < 0.05), but no effect of treatment. The BMD and TMD of the cortical bone was increased at 3 MO compared to D21 (*p* < 0.0001), but there was no significant effect of treatment, time, or the interaction between the two variables in either the cortical area or length. In the trabecular bone, there was a significant decrease in bone volume/total volume (*p* < 0.0001), trabecular number (*p* < 0.0001), BMD (*p* < 0.0001), and connectivity density (*p* < 0.0001), a significant increase in the spacing of the trabeculae (*p* < 0.0001) and TMD (*p* < 0.0001), and a tendency for an increase in trabecular thickness (*p* < 0.1) at 3 MO compared to D21. There was a tendency for an interaction between time and treatment in trabecular number (*p* < 0.1), as well as a tendency for an increased trabecular number at D21 and decreased at 3 MO in the FLX animals compared to the controls (*p* < 0.1). Finally, there was an overall effect of the interaction between time and treatment on the trabecular TMD (*p* < 0.1).

**TABLE 2:** Cortical and trabecular bone parameters evaluated by micro‐CT in gestation only animals.

		Measurements				*p* Value		
		D21		3 MO				
		CTRL	FLX	CTRL	FLX	Time	Treatment	Interaction
Cortical	Cortical thickness (mm)	0.109 ± 0.012	0.118 ± 0.013	0.177 ± 0.009	0.180 ± 0.006	<0.0001	0.0884	0.4677
	Periosteal perimeter (mm)	8.962 ± 0.176	8.680 ± 0.284	8.313 ± 0.232	8.391 ± 0.188	<0.0001	0.2372	0.0412
	Ct.ar (mm^2^)	1.728 ± 0.049	1.678 ± 0.070	1.726 ± 0.082	1.743 ± 0.086	0.2676	0.5667	0.2512
	BMD (mg Hg/cm^3^)	359.005 ± 41.032	385.074 ± 37.731	564.374 ± 28.335	575.553 ± 15.204	<0.0001	0.1094	0.5136
	TMD (mg Hg/cm^3^)	1168.569 ± 20.824	1167.185 ± 8.742	1257.577 ± 18.975	1246.656 ± 7.736	<0.0001	0.2594	0.3798
	Length (mm)	15.775 ± 0.187	15.525 ± 0.238	15.759 ± 0.322	15.896 ± 0.415	0.1671	0.6532	0.1331
Trabecular	BV/TV (%)	3.774 ± 0.995	4.133 ± 0.473	1.839 ± 0.586	1.737 ± 0.399	<0.0001	0.5650	0.3042
	Tb.N. (1/mm)	3.340 ± 0.285	3.582 ± 0.133	2.455 ± 0.142	2.446 ± 0.183	<0.0001	0.0903	0.0710
	Tb.Sp. (mm)	0.301 ± 0.028	0.279 ± 0.010	0.407 ± 0.020	0.408 ± 0.030	<0.0001	0.2380	0.1805
	Tb.Th. (mm)	0.030 ± 0.0014	0.031 ± 0.0017	0.034 ± 0.0044	0.032 ± 0.0045	0.0402	0.5987	0.2068
	BMD (mg Hg/cm^3^)	70.761 ± 9.858	74.558 ± 5.842	48.010 ± 7.235	49.192 ± 5.000	<0.0001	0.3306	0.6072
	TMD (mg Hg/cm^3^)	935.105 ± 24.428	940.050 ± 25.944	1007.166 ± 10.770	985.848 ± 9.051	<0.0001	0.2277	0.0580
	Conn. Density (1/mm^3^)	78.430 ± 32.153	81.898 ± 20.249	19.817 ± 6.734	19.564 ± 7.663	<0.0001	0.8006	0.7701

*Note*: Dams were treated throughout gestation only and harvested at the end of lactation (D21) (*n* = 5 CTRL; *n* = 8 FLX) or 3 months postweaning (3 MO) (*n* = 9 CTRL; *n* = 9 FLX). Data are presented as mean ± SD and analyzed using two‐way ANOVA for treatment and time. *p* < 0.05 is considered significant and 0.1 < *p* < 0.05 is considered a tendency.

Abbreviations: BMD, bone mineral density; BV/TV, bone volume fraction; Conn. density, connectivity density; Ct.ar, cortical area; Tb.N., trabecular number; Tb.Sp., trabecular spacing; Tb.Th., trabecular thickness; TMD, tissue mineral density.

### 3.2 Fluoxetine administration during gestation only alters maternal body composition, but does not affect litter size or circulating serotonin at the end of gestation

**FIGURE 3:**
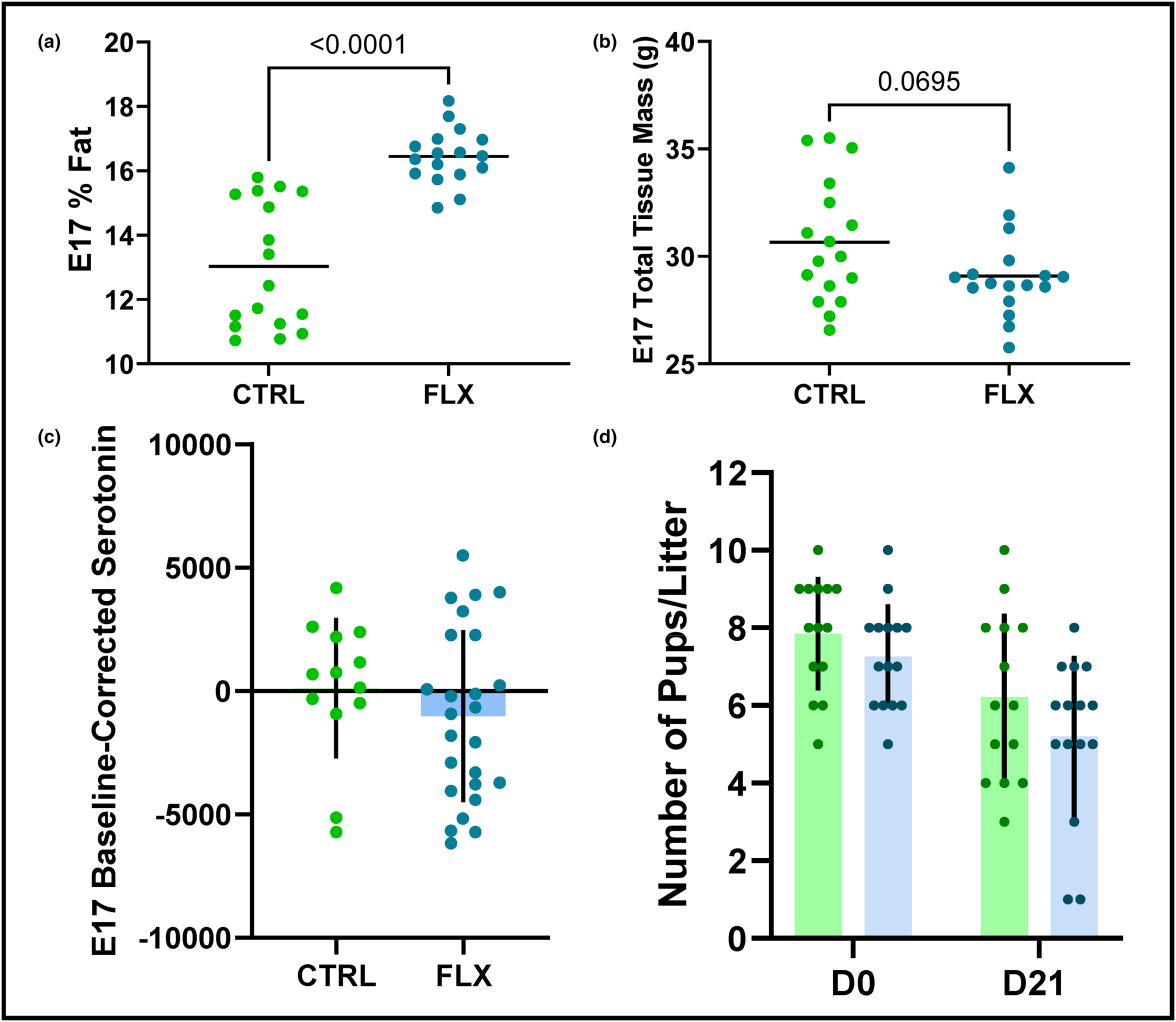
Fluoxetine during gestation only does not affect litter size, maternal circulating serotonin, or maternal weight gain. C57BL/6J dams were administered sterile saline (*n* = 16) or 2 mg/kg fluoxetine (*n* = 17) daily from the beginning of gestation through the end of pregnancy. (a) The percent body fat of the dams was measured via DXA at the end of gestation (E17). (b) The total tissue mass (TTM) was measured via DXA at the end of gestation (E17). (c) The circulating serotonin was measured at the end of gestation (E17). The circulating serotonin at E0 was used as the baseline to measure the change in circulating maternal serotonin. (d) The number of pups per litter was measured at the beginning and end of lactation. *p* < 0.05 is considered significant and 0.1 < *p* < 0.05 is considered a tendency.

The body composition of the dams was evaluated via DXA to determine the effects of gestational fluoxetine administration at the end of pregnancy. The FLX dams had a higher percent body fat than the control dams (*p* < 0.0001), but tended to have less total tissue mass (*p* < 0.1) (Figure 3a,b). In order to evaluate whether 2 mg/kg fluoxetine had an effect on litter size or pup mortality, the number of pups in each litter was measured at birth and again at the end of lactation. There were no significant differences in litter size at either the beginning or end of lactation (Figure 3c). Next, the impact of fluoxetine administration on circulating maternal serotonin was evaluated. Circulating serotonin was measured at the beginning and end of gestation. There was no difference in the change in circulating maternal serotonin at the end of gestation when normalized to E0 (Figure 3d). The weight of each dam was measured daily, and there was no difference between the change in dam weight relative to E0 between treatment groups.

### 3.3 During lactation only, fluoxetine administration has short‐term and long‐term impacts on bone

**FIGURE 4:**
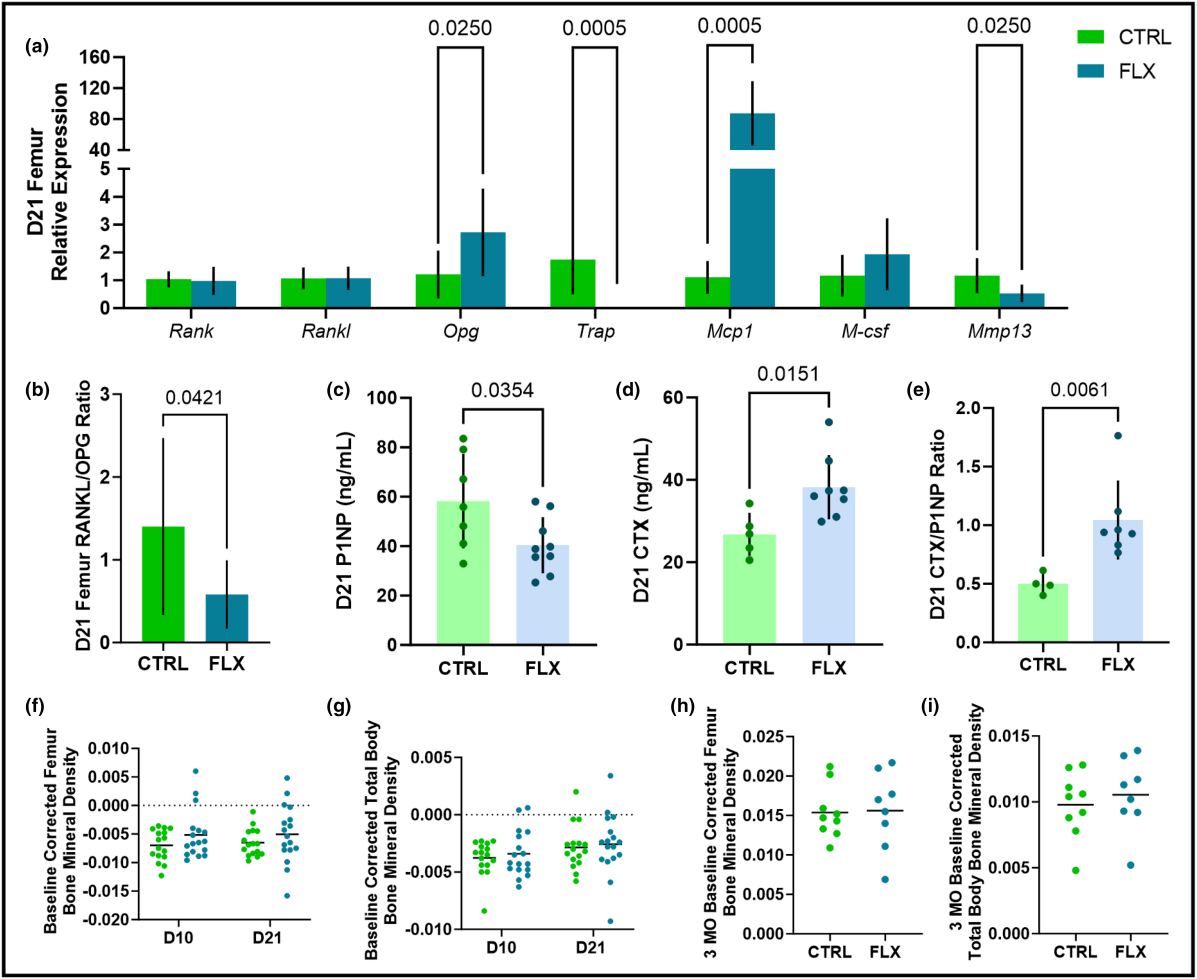
Fluoxetine administration during lactation only has an impact on markers of bone remodeling but does not impact short‐ or long‐term changes in BMD. C57BL/6J dams were administered sterile saline (*n* = 17) or 2 mg/kg fluoxetine (*n* = 18) from the beginning of lactation (D0) through the end of lactation (D21). (a) Femurs were harvested from the dams at D21 (*n* = 8 CTRL; *n* = 10 FLX), and the relative gene expression was measured. (b) In order to evaluate bone turnover in the femur, the *Rankl/Opg* relative gene expression ratio was measured. To further determine bone turnover, circulating concentrations of (c) P1NP, (d) CTX, and (e) the ratio of CTX/P1NP were measured. At D10 and D21, the BMD of the femur (f) and the total body (g) were measured and evaluated relative to a baseline measurement taken at the beginning of lactation (D2). Dams were aged to 3 months postweaning (*n* = 9 CTRL; *n* = 8 FLX) and the BMD of the femur (h) and the total body (i) were measured and evaluated relative to the measurement taken at the end of lactation to determine the change in BMD postweaning. *p* < 0.05 is considered significant and 0.1 < *p* < 0.05 is considered a tendency.

In the femur, gene expression was measured at weaning in the dams that were treated during lactation only. The gene expression of the RANK/RANKL/OPG pathway was measured, along with the expression of genes relevant to bone resorption (*Trap*, *Mcp1*, and *M‐csf*) and *Mmp13*, which is relevant to bone formation (Figure 4a). There were no differences in expression of *Rank* or *Rankl*, but expression of *Opg* was upregulated in the FLX dams (*p* < 0.05). Expression of Trap was downregulated in FLX dams (*p* < 0.001), whereas *Mcp1* was upregulated (*p* < 0.001) and *M‐csf* was unaffected. The expression of *Mmp13* was significantly downregulated in the FLX dams as well. To look at the ratio of bone breakdown to bone building in terms of gene expression at the level of the femur, the *Rankl/Opg* ratio was evaluated and was significantly decreased in the FLX animals (*p* < 0.05) (Figure 4b). To further examine bone turnover in the dams treated during lactation only, circulating P1NP, a marker of bone formation, and circulating CTX, a marker of bone breakdown, were measured in the serum at weaning (Figure 4c,d). Serum levels of P1NP were decreased (*p* < 0.05) and CTX was increased (*p* < 0.05) in the FLX animals. The ratio of circulating markers of bone formation to bone breakdown was measured via the CTX/P1NP ratio, which was increased in the FLX animals (*p* < 0.01) (Figure 4e). The changes in BMD of the femur and the total body relative to the BMD at the beginning of lactation (D2) were examined at peak lactation (D10) and at weaning (D21) (Figure 4f,g). There were no significant differences between the groups at either site or timepoint. Further, the BMD of the femur and total body was measured 3 months postweaning (3 MO) relative to the end of lactation (D21) (Figure 4h,i), where there was similarly no significant difference between the treatment groups at either site 3 months postweaning.

To further examine the short‐ and long‐term impacts of fluoxetine administration during lactation only on the maternal skeleton, micro‐CT was performed on the femurs of the dams at weaning and 3 months postweaning (Table 3). In the cortical bone, there was a significant increase in cortical thickness (*p* < 0.0001), BMD (*p* < 0.0001), TMD (*p* < 0.0001), and length (p < 0.0001) in the 3 MO cohort compared to the D21, and there was a tendency for an increase in cortical area (*p* < 0.1). There was also a significant decrease in the periosteal perimeter (*p* < 0.05) associated with age. In the trabecular bone, there was a decrease in BV/TV (*p* < 0.0001), trabecular number (*p* < 0.0001), BMD (*p* < 0.0001), and connectivity density (*p* < 0.0001) associated with age. Further, there was also a significant increase in trabecular spacing (*p* < 0.0001) and TMD (*p* < 0.0001) in the 3 MO animals compared to the controls. There was also a significant association between FLX treatment and decreased TMD (*p* < 0.001).

**TABLE 3:** Cortical and trabecular bone parameters evaluated by micro‐CT in lactation only animals.

		Measurements				*p* Value		
		D21		3 MO				
		CTRL	FLX	CTRL	FLX	Time	Treatment	Interaction
Cortical	Cortical thickness (mm)	0.139 ± 0.007	0.134 ± 0.009	0.184 ± 0.007	0.183 ± 0.009	<0.0001	0.3204	0.3648
	Periosteal perimeter (mm)	8.910 ± 0.331	8.626 ± 0.259	8.527 ± 0.166	8.509 ± 0.387	0.0201	0.1477	0.2020
	Ct.ar (mm^2^)	1.778 ± 0.073	1.700 ± 0.086	1.803 ± 0.067	1.788 ± 0.113	0.0603	0.1188	0.2946
	BMD (mg Hg/cm^3^)	444.601 ± 19.461	434.958 ± 26.018	571.139 ± 14.664	575.739 ± 23.739	<0.0001	0.7328	0.3381
	TMD (mg Hg/cm^3^)	1198.174 ± 13.552	1192.121 ± 10.521	1244.478 ± 13.534	1244.802 ± 7.069	<0.0001	0.4686	0.4202
	Length (mm)	15.716 ± 0.196	15.498 ± 0.406	16.059 ± 0.380	16.169 ± 0.150	<0.0001	0.6258	0.1471
Trabecular	BV/TV (%)	5.904 ± 1.389	5.275 ± 1.448	2.369 ± 0.731	2.230 ± 1.031	<0.0001	0.3506	0.5497
	Tb.N. (1/mm)	3.644 ± 0.198	3.585 ± 0.156	2.432 ± 0.180	2.570 ± 0.296	<0.0001	0.5908	0.1840
	Tb.Sp. (mm)	0.275 ± 0.016	0.279 ± 0.012	0.412 ± 0.035	0.391 ± 0.046	<0.0001	0.4285	0.2297
	Tb.Th. (mm)	0.035 ± 0.003	0.034 ± 0.0019	0.034 ± 0.012	0.033 ± 0.0029	0.7090	0.5193	0.8819
	BMD (mg Hg/cm^3^)	94.309 ± 15.316	88.149 ± 16.822	55.799 ± 8.583	54.848 ± 13.475	<0.0001	0.4591	0.5868
	TMD (mg Hg/cm^3^)	970.853 ± 14.275	952.240 ± 14.927	1005.690 ± 14.019	988.056 ± 18.562	<0.0001	0.0016	0.9263
	Conn. Density (1/mm^3^)	115.641 ± 26.918	117.203 ± 54.107	23.056 ± 10.288	28.172 ± 17.033	<0.0001	0.7695	0.8760

*Note*: Dams were treated throughout lactation only and harvested at the end of lactation (D21) (*n* = 8 CTRL; *n* = 10 FLX) or 3 months postweaning (3 MO) (*n* = 9 CTRL; *n* = 8 FLX). Data are presented as mean ± SD and analyzed using two‐way ANOVA for treatment and time. *p* < 0.05 is considered significant and 0.1 < *p* < 0.05 is considered a tendency.

Abbreviations: BMD, bone mineral density; BV/TV, bone volume fraction; Conn. density, connectivity density; Ct.ar, cortical area; Tb.N., trabecular number; Tb.Sp., trabecular spacing; Tb.Th., trabecular thickness; TMD, tissue mineral density.

### 3.4 Fluoxetine administration during lactation only impacted relative gene expression in the mammary gland, but did not impact circulating serotonin or calcium

**FIGURE 5:**
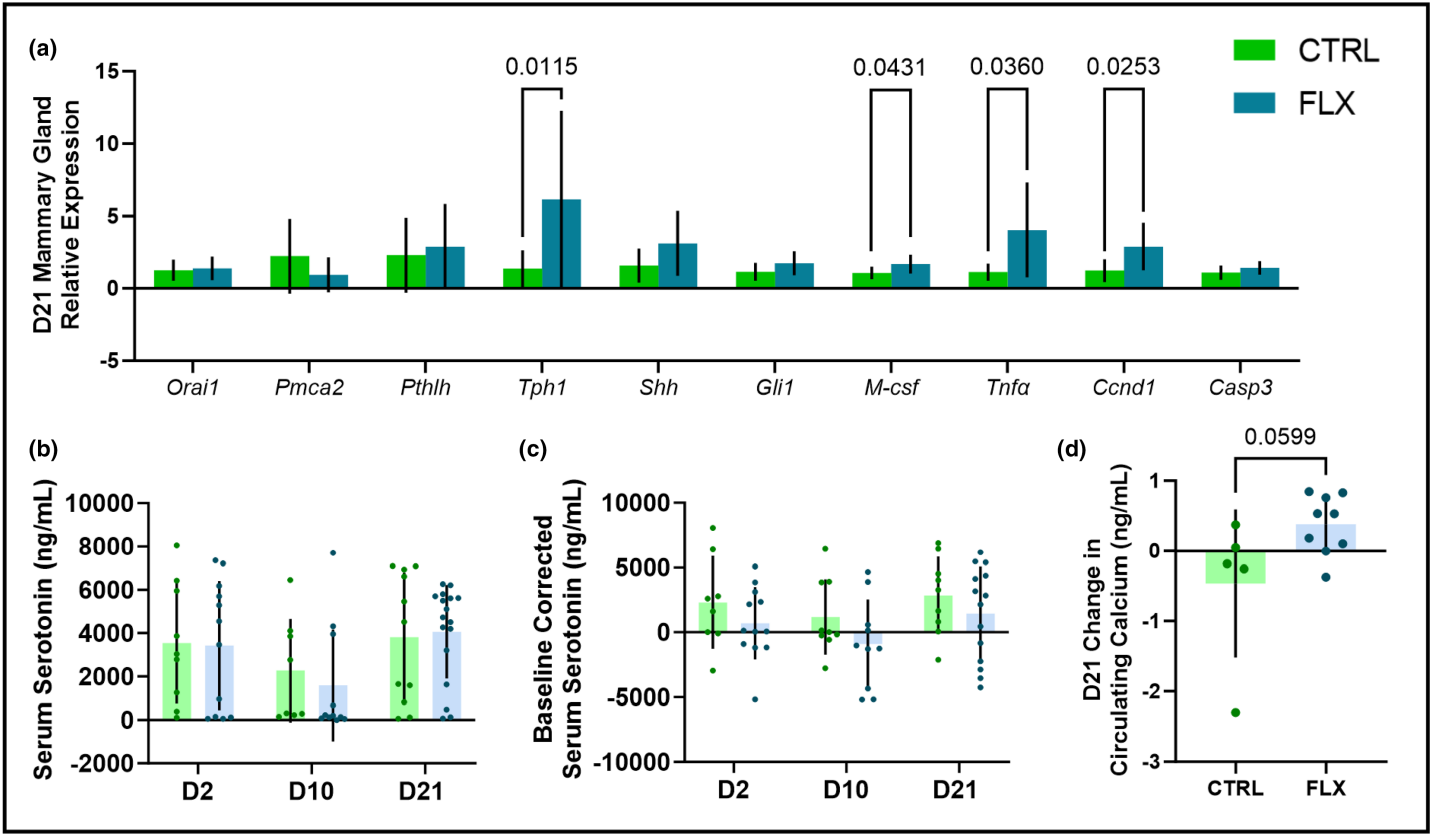
Fluoxetine administration during lactation only has effects on mammary gland relative gene expression, but not circulating serotonin or calcium. C57BL/6J dams were administered sterile saline (*n* = 17) or 2 mg/kg fluoxetine (*n* = 18) from the beginning of lactation (D0) through the end of lactation (D21). (a) A cohort of both the saline (*n* = 9) and fluoxetine (*n* = 8) mice were harvested at weaning, and the relative gene expression of the mammary gland was measured via RT‐PCR. (b) The circulating serotonin in the dams was measured at the beginning (D2), middle (D10), and end (D21) of lactation. (c) The circulating serum serotonin was measured relative to the circulating serotonin at the end of pregnancy (E17). (d) The circulating calcium at the end of lactation relative to the beginning of lactation. *p* < 0.05 is considered significant and 0.1 < *p* < 0.05 is considered a tendency.

To determine the effect of fluoxetine administration during lactation, the mammary glands of the dams were harvested at weaning, and the relative mRNA expression was measured (Figure 5a). There were no significant differences in expression of *Orai1* or *Pmca2*, a calcium channel and transporter, respectively. There was also no difference in expression of *Pthlh*, which encodes PTHrP. There was, however, an upregulation of *Tph1*, the rate‐liming enzyme in serotonin synthesis, in the FLX dams (*p* < 0.05). There were no differences in expression of *Shh* or *Gli1*, which are both important members of the serotonin‐dependent signaling pathway that modulates lactational bone remodeling. *M‐csf*, which is important in mammary gland remodeling and milk production during lactation, was upregulated in the FLX mice (*p* < 0.05). Further, *Tnfα* and *Ccnd1* were also upregulated in the FLX mice (*p* < 0.05 and *p* < 0.05, respectively). Both *Tnfα* and *Ccnd1* are associated with mammary epithelial proliferation. Finally, *Casp3*, which is associated with mammary involution, was unchanged. There was no difference in serum serotonin at any point during lactation, nor was there any change in circulating serotonin relative to the end of pregnancy between either group (Figure 5b,c). There was a tendency for there to be an increased change in calcium in the FLX dams at D21 compared to the beginning of lactation (*p* < 0.1), but it was not significantly different (Figure 5d).

### 3.5 When administered during gestation and lactation, fluoxetine has short‐term, but not long‐term impact on bone

**FIGURE 6:**
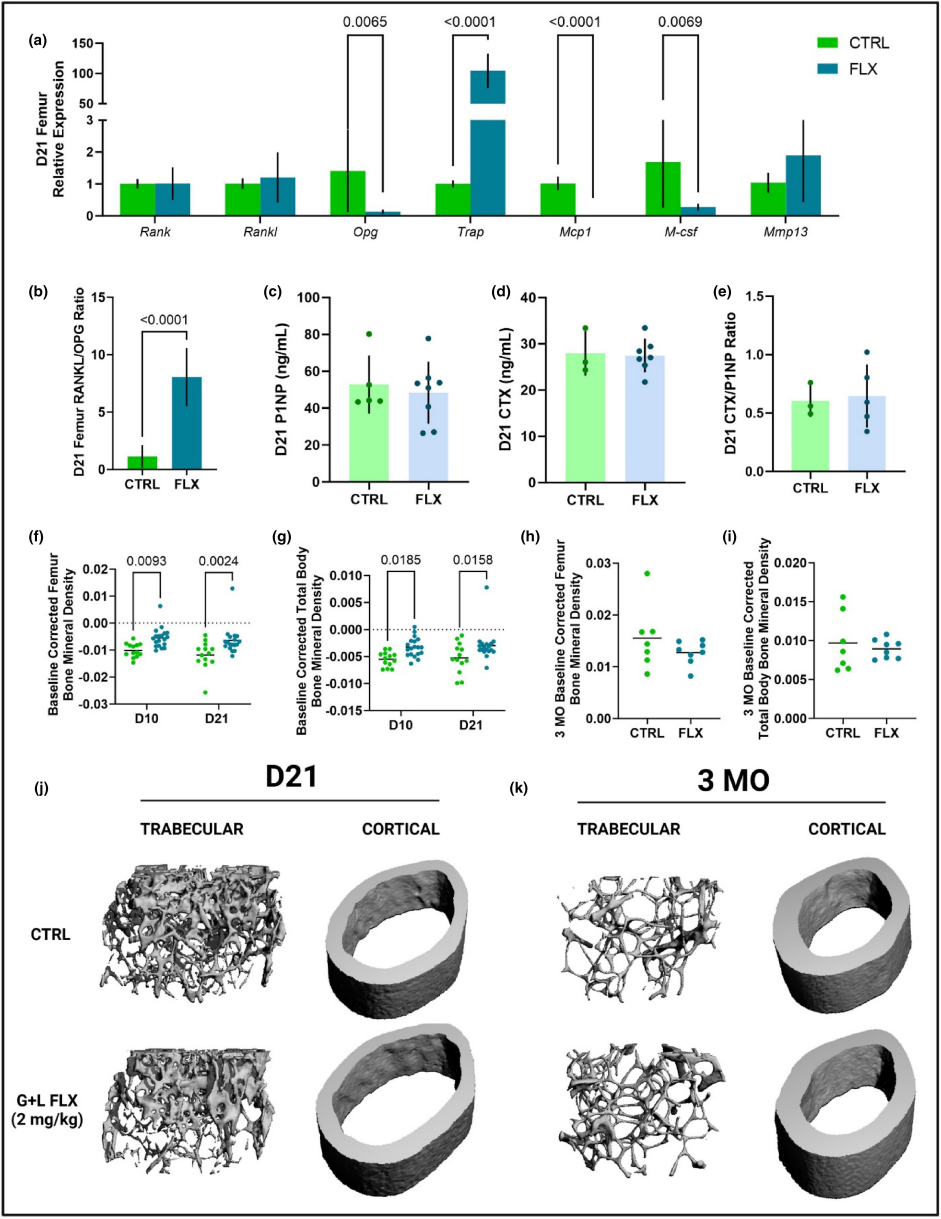
Fluoxetine administration during gestation and lactation has short‐term effects on bone turnover but does not have a long‐term impact on the change in BMD. C57BL/6J were dosed with either saline (*n* = 8) or 2 mg/kg fluoxetine (*n* = 10) during gestation and lactation. (a) At weaning, femurs were harvested and relative gene expression was measured. (b) The relative gene expression of RANKL/OPG was evaluated. (c) Circulating P1NP was measured at weaning. (d) Circulating CTX was measured at weaning. (e) The ratio of CTX/P1NP was evaluated. (f) The change in bone mineral density (BMD) of the femur relative to the beginning of lactation (D2) was measured. (g) The change in BMD of the total body relative to D2 was measured. (h) The change in BMD of the femur at 3 months postweaning (3 MO) relative to the end of lactation (D21) was measured in mice that were dosed with either saline (*n* = 8) or 2 mg/kg fluoxetine (*n* = 9). (i) The change in BMD of the total body relative to D21 was measured. (j) Representative images of femoral trabecular and cortical bone at D21. (k) Representative images of femoral trabecular and cortical bone at 3 MO. *p* < 0.05 is considered significant and 0.1 < *p* < 0.05 is considered a tendency.

In the femur, gene expression was measured at weaning in the dams that were treated during both gestation and lactation. The gene expression of the RANK/RANKL/OPG pathway were measured, along with expression of genes relevant to bone resorption (*Trap*, *Mcp1*, and *M‐csf*) and bone formation (*Mmp13*) (Figure 6a). There were no significant differences in expression of *Rank* or *Rankl*; however, expression of *Opg* was downregulated in the FLX animals compared to the controls (*p* < 0.01). Further, *Trap* was highly upregulated in the FLX animals (*p* < 0.0001), whereas *Mcp1* and *M‐csf* were downregulated (*p* < 0.0001 and *p* < 0.01, respectively). There was no significant difference in *Mmp13* expression. There was a significant increase in the *Rankl/Opg* ratio in the FLX animals compared to the controls (*p* < 0.0001) (Figure 6b). Interestingly, there were no significant differences in circulating P1NP, CTX, or in the P1NP/CTX ratio between treatment groups (Figure 6c–e). The change in BMD of the femur and the total body were measured throughout lactation relative to D2 (Figure 6f,g). The change in BMD of the femur was significantly increased in the FLX animals at both D10 (*p* < 0.01) and D21 (*p* < 0.01). Similarly, the change in BMD of the total body was also increased at D10 (*p* < 0.05) and D21 (*p* < 0.05). To determine the long‐term effect of 2 mg/kg FLX during gestation and lactation, the change in BMD at 3 months postweaning relative to the end of lactation was measured (Figure 6h,i). There were no significant differences in the change in BMD at 3 months postweaning between treatment groups at either site.

To analyze the effect of fluoxetine administration during both gestation and lactation on the maternal skeleton, micro‐CT was performed on the femurs of the dams at both D21 and 3 months postweaning (Table 4). In the cortical bone, there is a significant increase in cortical thickness (*p* < 0.0001), cortical area (*p* < 0.01), BMD (*p* < 0.0001), TMD (*p* < 0.0001), and length (*p* < 0.0001) between the D21 and 3 MO groups, and a significant decrease in the periosteal perimeter (*p* < 0.01) also associated with time. However, there are no significant differences in terms of treatment or the interaction between treatment and time in the cortical bone. In the trabecular bone, there is a significant increase in trabecular spacing (*p* < 0.0001) and TMD (*p* < 0.0001) between the two time points, as well as a significant decrease in BV/TV (*p* < 0.0001), trabecular number (*p* < 0.0001), BMD (*p* < 0.0001), and connectivity density (*p* < 0.0001). Further, there was a significant increase in TMD (*p* < 0.001) in the FLX dams at D21, along with a significant interaction between time and treatment (*p* < 0.01).

**TABLE 4:** Cortical and trabecular bone parameters evaluated by micro‐CT in gestation + lactation animals.

		Measurements				*p* Value		
		D21		3 MO				
		CTRL	FLX	CTRL	FLX	Time	Treatment	Interaction
Cortical	Cortical thickness (mm)	0.123 ± 0.006	0.129 ± 0.012	0.181 ± 0.011	0.179 ± 0.007	<0.0001	0.6576	0.2744
	Periosteal perimeter (mm)	8.776 ± 0.173	8.757 ± 0.214	8.530 ± 0.253	8.502 ± 0.256	0.0045	0.7756	0.9553
	Ct.ar (mm^2^)	1.716 ± 0.044	1.712 ± 0.064	1.775 ± 0.063	1.783 ± 0.080	0.0091	0.9474	0.7992
	BMD (mg Hg/cm^3^)	402.106 ± 20.404	420.092 ± 37.644	572.312 ± 26.225	564.227 ± 28.859	<0.0001	0.6505	0.2379
	TMD (mg Hg/cm^3^)	1186.936 ± 17.214	1195.630 ± 16.698	1246.813 ± 14.378	1249.857 ± 9.280	<0.0001	0.2765	0.5974
	Length (mm)	15.533 ± 0.356	15.478 ± 0.175	16.084 ± 0.347	15.913 ± 0.246	<0.0001	0.2872	0.5823
Trabecular	BV/TV (%)	4.889 ± 0.805	5.400 ± 1.180	1.819 ± 0.586	2.104 ± 0.280	<0.0001	0.1872	0.7039
	Tb.N. (1/mm)	3.519 ± 0.290	3.663 ± 0.170	2.459 ± 0.350	2.454 ± 0.154	<0.0001	0.4331	0.3983
	Tb.Sp. (mm)	0.286 ± 0.024	0.272 ± 0.013	0.407 ± 0.055	0.406 ± 0.026	<0.0001	0.5209	0.6164
	Tb.Th. (mm)	0.033 ± 0.003	0.035 ± 0.0019	0.035 ± 0.012	0.034 ± 0.0029	0.8091	0.9829	0.2202
	BMD (mg Hg/cm^3^)	83.218 ± 8.386	87.710 ± 12.218	47.378 ± 7.405	52.147 ± 3.478	<0.0001	0.1530	0.9653
	TMD (mg Hg/cm^3^)	930.282 ± 7.8 04	958.116 ± 14.684	994.010 ± 17.021	997.045 ± 5.369	<0.0001	0.0015	0.0088
	Conn. Density (1/mm^3^)	102.743 ± 24.728	107.475 ± 27.471	18.043 ± 15.941	26.014 ± 7.233	<0.0001	0.4046	0.8307

*Note*: Dams were treated throughout gestation and lactation and harvested at the end of lactation (D21) (*n* = 7 CTRL; *n* = 10 FLX) or 3 months postweaning (3 MO) (*n* = 7 CTRL; *n* = 8 FLX). Data are presented as mean ± SD and analyzed using two‐way ANOVA for treatment and time. *p* < 0.05 is considered significant and 0.1 < *p* < 0.05 is considered a tendency.

Abbreviations: Ct.ar, cortical area; BMD, bone mineral density; TMD, tissue mineral density; BV/TV, bone volume fraction; Tb.N., trabecular number; Tb.Sp., trabecular spacing; Tb.Th., trabecular thickness; Conn. density, connectivity density.

### 3.6 During gestation and lactation, fluoxetine affected serotonin at peak lactation and altered gene expression in the mammary gland at weaning

**FIGURE 7:**
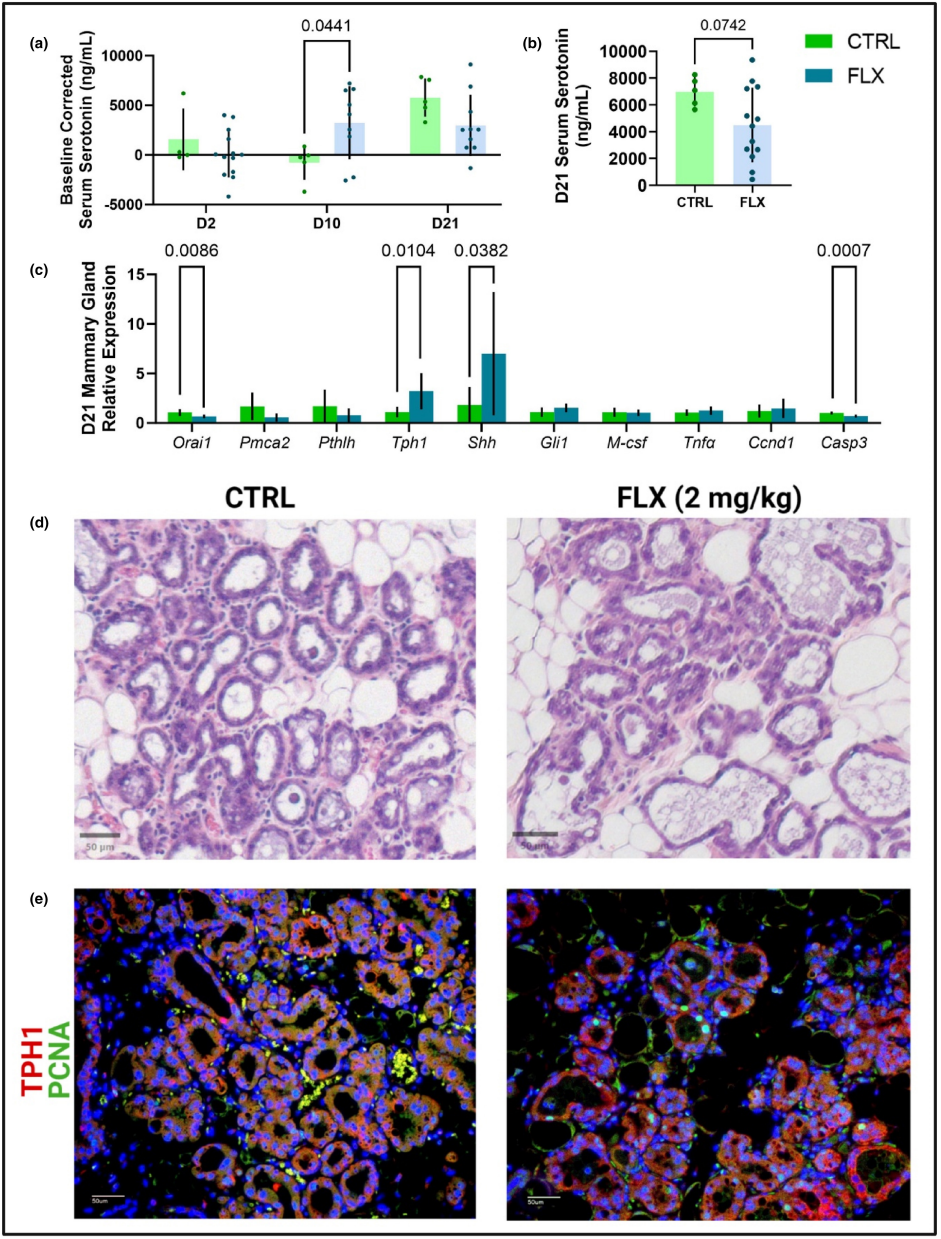
Peripartal fluoxetine administration impacts serotonin at peak lactation and mammary gland characteristics and gene expression at weaning. Dams were treated with saline (*n* = 14) or 2 mg/kg fluoxetine (*n* = 18) throughout gestation and lactation. (a) The circulating serotonin at the beginning (D2), middle (D10), and end (D21) of lactation relative to the circulating serotonin at the end of pregnancy (E17) was measured. (b) The circulating serotonin concentrations were measured at the end of lactation. (c) At D21, a cohort of dams were harvested at the end of lactation (D21) (*n* = 7 CTRL; *n* = 10 FLX). The inguinal mammary gland was collected and gene expression was measured via RT‐PCR. (d) Sections of the mammary gland were via H&E staining. (e) Tissues were further evaluated with immunofluorescence to visualize the expression of TPH1 and PCNA. *p* < 0.05 is considered significant and 0.1 < *p* < 0.05 is considered a tendency.

To explore the role of antidepressant usage during the peripartal period on the mammary gland, circulating serotonin, calcium, and the relative gene expression in the mammary gland at weaning were measured. Serotonin throughout lactation was measured relative to the end of pregnancy (E17) (Figure 7a). There were no differences at the beginning or end of lactation, but the FLX‐treated dams had a significantly greater increase in circulating serotonin compared to the controls at peak lactation (*p* < 0.05). There was no significant difference in the circulating serotonin at the end of lactation, but the FLX dams tended to have a decreased circulating serotonin compared to the control dams (*p* < 0.1) (Figure 7b). In the mammary gland, the calcium channel *Orai1* was downregulated in the FLX mice (*p* < 0.01) but there was no difference in the calcium transporter *Pmca2* (Figure 7c). Both *Tph1* and *Shh* were upregulated in the FLX mice (*p* < 0.05 and *p* < 0.05, respectively), but *Pthlh* remained unaffected. Finally, *Casp3* was downregulated in the FLX dams compared to the control dams (*p* < 0.001). The mammary gland was further examined via visualization with H&E staining and immunofluorescence (Figure 7d,e). The 2 mg/kg group has more characteristics of involution progression than the control group. Further, there is visually less TPH1 expression and more PCNA expression, the tissue contains more adipose, and the alveoli appear to be fuller.

## 4 DISCUSSION

In this study, we have demonstrated that administration of a low dose of fluoxetine has differential effects on the dams based on the window of administration. Overall, there were minimal effects on bone at weaning and 3 months postpartum in the dams that were exposed during gestation only. In humans, 80% of the calcium accrual in the fetal skeleton happens during the third trimester, and in rodents, the majority of fetal skeletal mineralization happens in the last few days of gestation (Comar, 1956; Givens & Macy, 1933). Pregnancy is unique because calcium absorption in the maternal body doubles, which is not the case during lactation (Shenolikar, 1970). Because calcium absorption is increased during this time period, the maternal skeleton does not have to participate in supplying calcium to the same extent as it does during lactation, and so the minimal effect of fluoxetine on the skeleton is not surprising. Additionally, these dams did not exhibit decreases in serum serotonin, which we have observed in a previous study where maternal bone was decreased (Weaver et al., 2018). Administration of a low dose of fluoxetine had no effect on the change in dam weight during gestation, nor did it have an effect on the number of pups born or the number of pups that survived until weaning. Based on the lack of a change in serum serotonin concentrations, this is not surprising. In previous experiments, we have used a high dose of fluoxetine (20 mg/kg). When taking physiological differences such as blood volume, blood turnover, and body surface area, the high dose of fluoxetine is comparable to a 60 kg human taking 90 mg a day, whereas the dose used in the present study (2 mg/kg) is comparable to a 10 mg daily dose. We have reported that a high dose of fluoxetine (20 mg/kg) administered during gestation decreases maternal prepartal weight gain and decreases the number of pups born per litter (Domingues et al., 2022). This, however, was not observed with the low dose of fluoxetine. It appears that the 2 mg/kg dose of fluoxetine did not reduce serotonin concentrations until the end of lactation. However, it can be difficult to draw conclusions fully due to the long half‐life of fluoxetine. In humans, fluoxetine has a half‐life of 1–3 days and its active metabolite, norfluoxetine has a far longer half‐life of 7–15 days (Lemberger et al., 1985). The half‐life of fluoxetine and norfluoxetine is considerably shorter in rodents due to their rapid metabolism, but the overlap between the beginning of lactation and circulating fluoxetine and norfluoxetine after the cessation of treatment must be acknowledged.

In the dams exposed during lactation, there were alterations in femur gene expression, as well as markers of bone remodeling. Despite this, there were minimal effects on the cortical and trabecular bone at weaning and 3 months postweaning. During lactation, calcium absorption is the same as during a homeostatic state, and so most of the calcium provided to the offspring is sourced from the skeleton (Kovacs, 2016; Specker et al., 1994). However, this is specific to humans, as rodents experience an increased intestinal transport of calcium that peaks during lactation (Halloran & DeLuca, 1980). One explanation for this physiological difference may be the intense demand for rodents, given the short duration of lactation and the number of offspring they have as a litter‐bearing species. Though there were very minimal lasting effects on the maternal skeleton, there was evidence that the low dose of fluoxetine modulated bone remodeling during lactation. The RANKL/OPG ratio was decreased at weaning, but P1NP, a marker of bone formation, was decreased in the fluoxetine‐treated dams, and CTX, a marker of bone breakdown, was increased. Further, the ratio of CTX/P1NP was increased in the fluoxetine dams, which indicates that bone‐building activity was decreased at the end of lactation, while bone resorption was increased. In the dams dosed throughout the entire peripartal period, there was also altered gene expression of the femur, but the changes in circulating markers of bone remodeling were not observed. There were minimal short‐term or long‐term effects on the cortical or trabecular bone. In contrast to the lactation‐only dams, there was an eight‐fold increase in the expression of RANKL to OPG, which indicates bone remodeling. Interestingly, there were no significant differences in circulating P1NP, CTX, or the CTX/P1NP ratio, but there was a significant increase in the baseline‐corrected femur BMD and total body BMD at both peak lactation and the end of lactation. This effect did not persist 3 months postweaning in either a whole body or site‐specific context. Interestingly, the dams treated with 2 mg/kg FLX did not have decreased serum serotonin concentrations until weaning. This contrasts with what we observed previously in dams treated the entire peripartal period at 20 mg/kg FLX (Weaver et al., 2018) Typically, decreases in serum serotonin are observed once SSRIs have reached their effective dosing, which may explain the phenotypic differences observed between the high and low doses. It is likely that at the lower dose of 2 mg/kg of fluoxetine, it takes longer to have an impact on the mammary gland and bone crosstalk.

When considering the effects of fluoxetine administration, specifically on bone, the length of dosing is important. Of note, Ortuño and colleagues demonstrated that administration of 20 mg/kg fluoxetine to male and nonpregnant, nonlactating female mice resulted in net bone loss in a chronic model of fluoxetine use; however, in an acute model, an antiresorptive effect was observed (Ortuño et al., 2016). The different dosing paradigms were 6 weeks and 3 weeks, respectively, which is significant to the present study due to the gestation and lactation period lengths in mice. C57BL/6J mice have a 19‐day gestation and a 21‐day lactation, so dosing during either gestation or lactation alone is much closer to the acute dosing paradigm, while dosing throughout the entire peripartal period is a similar length to the chronic model. Despite the antiresorptive effects seen in virgin females after being dosed with fluoxetine for 3 weeks, these results were not recapitulated in the lactation‐only dams. This could imply that the overall resorptive effect of lactation is greater than the antiresorptive effect of fluoxetine. Or, perhaps, it can be due in part to a dose effect. Ortuño and colleagues only used a 20 mg/kg dose of fluoxetine, as opposed to a 2 mg/kg dose, which could also be an explanation for why the circulating P1NP and CTX in our lactation‐only model suggest an overall resorptive effect on the skeleton. Restoration of the maternal skeleton postweaning is thought to occur via a few different mechanisms. Firstly, weaning triggers widespread osteoclast apoptosis, which decreases the expression of *Rankl*, which, in turn, decreases the RANKL/OPG ratio (Ardeshirpour et al., 2007). In homeostatic conditions, bone resorption activity is usually coupled with bone remodeling activity, but after weaning, despite the decrease in osteoclast activity, a decrease in bone formation is not observed, which hints at an uncoupling of bone formation and resorption that contributes to the restoration of the maternal skeleton postweaning (Wysolmerski, 2010). In both the lactation dams and the gestation and lactation dams, there was no significant difference in the expression of *Rankl*. Instead, *Opg* expression was what was primarily altered, which led to the differential RANKL/OPG ratio between the two fluoxetine‐treated groups at weaning.

In the mammary gland, there were alterations in gene expression in the lactation and the gestation and lactation animals, and changes in gene expression were different depending on whether the dams were dosed postpartally or peripartally. Finally, there was an effect on circulating serotonin at peak lactation in the animals dosed during pregnancy and lactation, but that was not recapitulated in the dams dosed during lactation only. In the dams that were only dosed during lactation, there was an upregulation of mammary gland *Tph1* expression. The dams that were dosed throughout the entire peripartal period also had upregulation of *Tph1* expression in the mammary gland, but the change in circulating serotonin was increased at peak lactation. Interestingly, the mammary glands in the dams treated in both gestation and lactation appear to have increased adipose and distended alveoli compared to the control treated dams. Additionally, it appears that there is more robust staining of PCNA in the control treated dams compared to dams treated with FLX. These data suggest that dams treated with SSRI could potentially undergo weaning more rapidly. This coincides with the decreased circulating serotonin concentrations that were observed at weaning only in these dams as well, an indicator of SSRI effectiveness. This is also consistent with previous studies indicating that increased SSRI treatment can break down tight junctions, leading to a hastening of mammary gland involution, even at a more modest dose of FLX (Marshall et al., 2010; Sheftel et al., 2022). However, the lack of changes in PTHRP and other calcium‐related genes could be due to the late decrease in circulating serotonin concentrations, despite observing increases in *Shh* gene expression. Our previous data in which we observed both increased *Shh* and *Pthlh* gene expression and reduced gene methylation were at higher doses of FLX that suppressed circulating serotonin concentrations much earlier, as well as much more robust induction of serotonin signaling (Laporta et al., 2014; Weaver et al., 2018). Peripheral serotonin in circulation is primarily taken up by platelets via SERT. Platelets do not contain TPH1 and therefore cannot produce serotonin, so inhibition of SERT with SSRIs results in the depletion of serotonin within platelets (Mauler et al., 2019). Platelet expression of SERT is regulated by plasma serotonin concentrations in a biphasic manner: increased serotonin in plasma initially results in increased SERT expression and serotonin uptake, but then is decreased with higher serotonin concentrations (Mercado & Kilic, 2010). Because of this, the effect on circulating serotonin during lactation may be partially due to the length of dosing, leading to more mild effects on the mammary gland. Our findings in the mammary gland would support the more diminished effects on the maternal skeleton.

There are several limitations of this study that warrant discussion. First, we did not examine different doses of fluoxetine, nor did we analyze any other SSRIs. Previously, we have conducted similar studies using a high dose of fluoxetine or sertraline, another member of the SSRI class of antidepressants, but these studies only included dosing during the entire peripartal period (Sheftel et al., 2022; Weaver et al., 2018). To separate out what SSRI, dose, or period of administration causes the greatest insult to the maternal skeleton, more studies must be conducted involving different doses of fluoxetine, different antidepressants, and different windows of exposure. When considering peripartal fluoxetine usage, dose may be an important consideration. In humans, fluoxetine has been shown to be effective at as little as 5 mg/day, but not at 60 mg/day, and so a low dose might be effective enough to mitigate the underlying psychiatric disorder that is being treated (Berney, 2005). A higher dose of fluoxetine may not even be necessary, as there is evidence that a high dose of 60 mg/day is less effective in the treatment of major depressive disorder (Beasley et al., 1990). Though the present study used a relatively low dose, it has been shown that administration of many SSRIs well below the lowest manufactured dose resulted in 50% SERT occupancy (Sørensen et al., 2022). In the present study, we were unable to generate sufficient animals with the high dose of fluoxetine due to the deleterious effects it has on pregnancy outcomes and pup mortality, and thus the lower dose was chosen (Domingues et al., 2022). The dose chosen for this study is also a very low dose when comparing circulating fluoxetine and norfluoxetine in humans taking the SSRI versus rodents (Dulawa et al., 2004). Further, the mammary gland was only analyzed at weaning, when milk production has decreased as the pups start eating solid food and involution is beginning. However, while only *Tph1* and *Shh* gene expression were increased unlike in previous studies, the mammary glands in the FLX‐treated dams appeared to have increased adipose tissue and reduced PCNA at weaning than controls. Future studies should focus on the mammary gland at different phases of lactation to evaluate the effect of fluoxetine more fully during the peripartal period at a lower dose.

In conclusion, a low dose of fluoxetine during gestation, lactation, or the peripartal period did not have long‐term structural effects on the dam skeleton, contrary to our previous work conducted with a high dose of fluoxetine (Weaver et al., 2018). This may be attributed to the differences in the present study such as the window of exposure and the lower fluoxetine dose administered. However, there were differential changes in bone remodeling observed in both the groups administered fluoxetine either during lactation or the entire peripartal period. At the level of the mammary gland, there were also differential changes in gene expression between these two groups as well. Fully elucidating the dam‐specific effects of peripartal SSRIs is of critical importance, as 6% of all pregnant individuals are exposed to an SSRI and the prevalence of postpartum depression is 10%–15% (Cooper et al., 2007; Kroska & Stowe, 2020). Determining the antidepressants and doses that are safest to use during the peripartal period is important for the well‐being of both members of the breastfeeding dyad, and investigating how antidepressants differentially affect maternal physiology depending on when they are administered will help doctors and their patients make the best‐informed decisions about treating psychiatric disorders during the peripartal period.

## AUTHOR CONTRIBUTIONS

H.P.F., J.F.C., and L.L.H. conceived of the experiments, analyzed, and interpreted the results. L.A.W. and J.F.C. analyzed bone micro‐CT data. C.J.K, M.J.P., M.A.R., and L.J.B. analyzed images and collected data. All authors read and approved manuscript.

## FUNDING INFORMATION

This work was funded by NICHD R01HD094759 to Laura L. Hernandez. Hannah P. Fricke was funded by a T32HD041921. Julia F. Charles is funded by NIAMS R01AG046257 and R21AR07768.

## CONFLICT OF INTEREST STATEMENT

The authors have nothing to disclose.

## ETHICS STATEMENT

All experiments were approved by the Research Animal Care and Use Committee at the University of Wisconsin–Madison and were performed under protocol number A005789‐R01‐A03.

## Data Availability

All data are presented in the manuscript and can be accessed upon request to authors.
